# Disease, Its Conditions and Treatment: An Inaugural Essay Submitted to the Medical Faculty of the University of Louisville, March 5, 1852, for the Degree of M. D.

**Published:** 1852-04

**Authors:** Waldo Lewis

**Affiliations:** Missouri; Louisville, Ky.


Art. III.—*Disease, its Conditions and Treatment:* An Inaugural Essay submitted to the Medical Faculty of the University of Louisville, March 5, 1852, for the degree of M. D. By Waldo Lewis, M. D., of Missouri.

There is a condition of the general system characterized by the perfect integrity of every structure, and the harmonious play of every function, and this is health. Any departure from this condition, whether attended by appreciable change of structure, or merely evidenced by altered functional action is disease. One is the physiological action of every part of the organic machinery; the other is the pathological action of one or more parts. Now, these definitions of the terms health and disease, though strictly scientific, are not practical; for, opposite as are these conditions in their nature, and strikingly different as are their essential features when presented in a marked or mature form; yet, so nicely is the one shaded off into the other, so intimate is association of their slight manifestations, and so rarely are they ever observed entirely dissevered, that, if the strict definition of health be rigidly maintained, it could not be said any individual is in its enjoyment. Hence, for practical purposes, it is assumed that health does not imply a perfect integrity of every organ, but simply a freedom from such structural changes as endanger life, by impeding the normal exercise of the vital functions. In this sense of the term, health is compatible with many permanent alterations of structure, as the loss of an arm, a leg, an eye, and even with the loss or derangement of one of the senses, as seeing, smelling, &c. Having thus established a clear and definite idea of health, it will not be difficult to understand the proper sense in which the term disease is used. Any departure from the normal or physiological condition, in which there exist such changes of structure as tend to destroy life by embarrassing vital action, constitutes disease. It is thus seen that there can be no standard condition of either, both being susceptible of innumerable variations. Now, what the essential nature of disease is, or what may be its proximate cause, is as inscrutable as the cause of gravitation, or chemical affinity; it is not attempted in this paper to solve the mystery; nor is it designed to investigate the nature and treatment of an hundredth part of the diseases elaborated in the books. My object is to throw out some plain, practical and it is hoped philosophic ideas of the conditions of the system under the various modifications, which have been noted and named in systematic treatises as particular and individual diseases. For, I conceive that the innumerable affections daily presenting themselves for observation, are but the varied forms of a certain definite condition, modified by a variety of circumstances, each varied form or modification having been recognized in the books as a particular disease, and designated by a particular name. And here let it be said, that I neither investigate nor treat diseases by name, but address both speculation and treatment directly and solely to the condition, whence springs the morbid action. There is, I humbly conceive, nothing in the wide scope of medical practice so fruitful of evil, so disastrous in consequences, as the common habit of naming diseases, and treating them accordingly. It shackles the mind of the practitioner with false ideas—it bewilders his judgment—it cramps his energies—it binds him the unconscious slave of routine empiricism, and makes him the instrument of irreparable injury rather than of lasting benefit. To the practical and philosophic physician, it matters not what may be the name applied to the disease engaging his attention; whether it be small pox or typhoid fever, croup or consumption, his sole aim is to ascertain and remove the condition of the system giving rise to, and evidenced by the presenting phenomena.

It has long been a question whether disease could exist independently of inflammation; in other words, whether there could, strictly speaking, be a "functional disease." I do not presume to discuss the subject; it is too vast and intricate for my limited powers. But in the present unsettled state of the question, the most satisfactory ideas are, perhaps, those thrown out by Professor Gross in the preliminary chapter of his Pathological Anatomy. Using the term organic disease to denote not only permanent changes of structure, but also every temporary alteration which the tissues experience when in a state of disease, he says: "It may perhaps be doubted whether under any circumstances there can strictly speaking, be a functional disease; or in other words a mere aberration of the physiological state of a part without some change in its anatomical elements; and it may be assumed as a proposition liable to few exceptions, that all organic diseases whatever be their seat or extent, are the result of inflammation either of an acute or chronic kind."

Assuming then this proposition, viz: *That all diseases arise from a change in the anatomical elements of some organ or tissue*, it remains to be considered whether this change be inflammation or irritation, and what may be the relation they sustain to each other. It is difficult, nay impossible, to prove such a definition of inflammation as shall strictly apply to all its varying forms, and always convey a clear idea of its presenting phenomena; and it is not less difficult to solve the mystery that envelops its intimate connexion with irritation, or trace the line where the one merges into the other. Yet there are certain striking differences, *not in kind, but in degree*, which it is all-important to discriminate. Inflammation when fully developed, is manifested by a series of well-marked and easily recognized phenomena: there is in the part affected, a change of color, an increase of temperature, pain and tumefaction; its action may be of great intensity and short duration leading to rapid disorganization of texture, or it may be mild in action and of long persistence, slowly changing normal structure. Inflammation induces great disorder of function—it tends to destroy texture—it terminates in death of the part or it resolves in health---effusion and suppuration not being here considered as terminations, but simply as attending results or stages of the inflammatory process. Inflammation cannot exist without being preceded by and attended with irritation; the one being the first appreciable step or movement toward the production of the other; on the contrary, irritation, though inseparable from inflammatory action, can exist independently of inflammation, at the same time its constant tendency being to intensify or pass into that condition. Like inflammation, its primary source is undoubtedly in the nervous system; but, unlike inflammation, irritation seldom confines itself to one organ or tissue, unless it be to intensify and merge into the former condition; and though ever present in greater or less degree in existing inflammation, irritation may spring up in some organ or tissue without any such apparent changes as discoloration, heat, pain or swelling, may implicate an entire system, and finally may pervade the entire system, affecting every tissue and deranging every function, circulatory, secretory, excretory or nutritive. But this condition of general irritation tends strongly toward the condition of inflammation, and in fact can persist for no great length of time without developing inflammatory action in some organ; not from any peculiar selective power inherent in irritation, but because the parts involved in inflammation, succumb to the morbid influence on account of inability to shake off the irritating power, its conservative forces being impaired by a variety of circumstances.

Now, no serious inflammation can exist in any part of the body without involving the whole in disturbance; the inflammatory action is limited to a certain extent of tissue, and it is constantly striving to transcend its limits; but a strong conservative power resisting its encroachments, it makes but slow progress; while beyond the immediate sphere of inflammation a mysterious influence is at work, invading organ after organ and tissue after tissue until all functional action is perverted, and the entire system thrown into commotion. This condition of general irritation constitutes what is called inflammatory fever, and as such a condition tends to the production of organic changes of a serious character, some part sensitive to the morbid influence from structure or function, or less fortified by the conservative power, gives way, and another inflammation is lighted up. This is metastatic inflammation; and it is in this manner that inflammation is extended—that is, by irritation, continuous, contiguous or sympathetic.

Upon these considerations then it is assumed, that inflammation is always preceded by irritation—is constantly attended by irritation, and may engender irritation in other and distant parts of the body, while irritation may arise entirely independent of any inflammatory action—may pervade every structure and eventually develop inflammation in some organ. Hence, between irritation and inflammation, the most intimate relation exists.

Bearing in mind then the fact that health or physiological action is directly dependent upon integrity of structure, the conclusion is inevitable, that any departure from physiological action wide or minnte, must be preceded and caused by a change in structural integrity—that lesions of function spring from lesions of structure—that they bear to each other the mutual relation of cause and effect; and consequently, that there can be no such condition as a purely "functional disease." The basis then is no longer tenable, on which rests the theory of an occult influence altering and perverting normal functional action in some mysterious way without changing structure.

Disease must consist essentially in a "change in anatomical elements." For it is impossible to conceive of an effect without a cause; perverted function is an abnormal condition, and it can only arise from an abnormal condition of structure. Assuming then that disease springs always from anatomical change, and that the primary or first appreciable effect of this change is irritation—subsequently inflammation—it would not appear illogical to consider disease as essentially a unit.

It is important, here, to have a clear understanding of the *sense* in which the terms inflammation and irritation are used in this paper, when expressing a single condition. The great condition, existing under every form of morbid action, is the condition of irritation and inflammation; the terms in *essence* are the same, differing only in degree. Yet, for obvious reasons, it is convenient to establish an arbitrary distinction, and consider them practically as two conditions, viz: modified in a thousand different ways—assuming innumerable Protean shapes—attacking now this structure and now that, hastening by rapid strides to destroy life, or lurking in a mysterious veil, pausing but to grow stronger; these conditions constitute the modes of morbid action recognized, named and treated in the books as different "diseases."

The general treatment of irritation and inflammation is too well known to require more than a passing notice. I pretend not to the discovery of any new therapeutic agents, or to be gifted with magic power in the administration of the old. As the nature of the two conditions is essentially identical, so the same general principles of treatment apply as well to one as to the other, modified only in degree. Special treatment, however, varies in accordance with the particular form under which the condition is manifested. The different phases of the condition modifying the general treatment to suit each separate form : in one case it is actively antiphlogistic—in another, tonic and stimulating—in a thi d, alterative or eutrophic, and so on; but in all cases and under all circumstances, the practitioner must have his constant attention fixed upon the condition at large, regardless of its particular name as presented in the form under consideration ; he must keep vigilant watch over the great organs of the body—he must beware of interposing his meddlesome hand when not required ; and he must be equally careful of withhodling his aid when indicated.

Before treating a patient it is necessary to find out what is the matter with him ; and here presents the greatest difficulty which is to be encountered. An error in diagnosis may be fatal, while a correct appreciation of the peculiar form the condition has assumed, at once sheds light upon the proper remedies to be employed for its removal. Now, to arrive at this great desideratum, a careful and thorough examination of the patient must be had ; his age, habits of life, occupation, previous history—in fine, a variety of particulars must be inquired into ; expression of countenance and position in bed are to be noted, and a scrutinizing examination of the entire body must be instituted, the contents of the great visceral cavities claiming first attention. It is needless here to particularize the precise manner in which this examination is to be conducted ; the judgment and adeptness of the practitioner is brought into active requisition in these preliminaries ; auscultation, percussion, palpation, succussion, and a variety of other means are to be employed. It may again be said, that upon the proper discharge of this important duty, depends mainly the subsequent result of the case.

As disease is considered as a *unit*, and the different affections named in the systematic treatises on practical medicine as but so many modifications of the condition; and as it would require volumes rather than pages, to contain even an outline of the treatment appropriate to each and every modification, it can only be attempted in this paper, to give a cursory sketch of the symptoms and management of a few individual forms.

The special nature or isolated peculiarity of certain forms of disease, is a subject too extensive and intricate to be herein discussed; in what their special character consists is a profound mystery; yet under the view of disease already advanced, this specificness may be regarded simply as a peculiar influence or principle, existing under certain peculiar circumstances, and modifying the condition in a definite manner.

Small pox, measles, and scarlatina, are treated conditionally, and hence philosophically, their special character influencing only precautionary or sanitary measures in reference to contagion. For syphilis on the contrary, the treatment is wholly empirical; experience alone having demonstrated the efficacy of nitrate of silver in primary chancre; of mercury and iodine in secondary, and of the salt of potassium in the tertiary form of the disease. A simple abrasion upon any part of the body will usually heal without any application at all; but a chancre or specific abraslon must be cauterized or it will empoison the system; yet they are both forms of the condition of inflammation, one existing under a peculiar and specific modification, and the other simply presenting the usual features.

It remains to speak of a form of the condition of irritation. An individual is taken sick, and demands medical assistance; the usual inquiries having been made, it is found that there has been no cause appreciable by the patient of his indisposition; he has been weary and languid for a day or two—has not felt like attending to business—has lost his appetite—is somewhat costive, but has noticed no unusual symptoms about his bowels; in short, has thought but little about the matter, hoping the indisposition would soon wear off. A proper examination of the body does not reveal any prominent symptom. There seems to be no abnormal condition of the brain, or of the thoracic and abdominal viscera—no pain is complained of, save a slight head-ache, and a sort of aching about the joints and in the lumbar region—pressure elicits no tenderness; and in short, there is no particular handle to take hold of; yet the tongue is a little coated—the external surface a little warmer and dryer than natural, and the pulse indicates a quickened circulation; a sort of uneasy, restless sensation is about all that troubles the patient. Now, what ails this patient, and what must be done to relieve his condition? A morbific impression has been made upon the nervous system, partial or general; the nerves being the regulating and controlling power, have resisted the impression for a time, but unable to withstand its influence, have called in aid; the blood-vessels responsive to the call hasten to the rescue, and presently the whole system is roused into activity to shake off the foe, and failing in this, a commotion is established; the conservative forces yield to the morbid influence, and the condition of general irritation is set up. This is called "fever;" but what fever is it? This is precisely the manner in which typhoid fever commences, or small pox, or bilious remittent fever, and many other affections; and it may prove to be any one of these diseases, just because it is a great general condition, susceptible of endless modifications. A knowledge of exposure to small pox contagion might lead to a rational opinion as to that disease; but in the absence of such knowledge who can tell what may eventuate? The philosophic physician pauses not to inquire for a suitable name, but premising the necessary attention to the immediate external circumstances of the patient's room, employing the pure air of heaven as a constant nurse at the bed-side, addresses his remedies to the existing condition. The indications call not for perturbing and active, but gentle and expectant treatment—the enemy must not be fought, but soothed and coaxed out of the premises. With this view, the bowels are gently moved with a simple purgative, of which calomel forms a part, (the peculiar powers of calomel cannot here be expatiated on,) and cooling drinks are ordered *ad libitum*. These may be, simple iced water, lemonade, tamarind water, spt. minder. and nitre, citrate of potassa, or any kindred article the fancy of the practitioner or taste of the patient may suggest. Interdicting all disturbing influences, the patient is left to quiet and convalescence, or to a further development of symptoms by which, when justly appreciated, all subsequent treatment is to be regulated. Should this general irritation persist and increase in intensity, notwithstanding the remedies, the great proclivity to merge into inflammation must be borne in mind, and a vigilant watch kept over the great organs of the body; should inflammation be set up, it must be promptly combated by appropriate antiphlogistic treatment. This untoward event sometimes occurs, and the inflammatory action is so completely masked, as to escape the strictest scrutiny; yet the physician must beware of presuming too much upon its existence in the absence of definite symptoms, and avoid pushing his treatment too far.

The great point is to husband the conservative powers, and not to weaken them by active medication in the vain hope of cutting short the disease. The practice of giving physic at random is much to be reprobated. If a careful consideration of existing symptoms does not lead to a satisfactory idea of the precise condition, it is far better to wait an issue than to risk doing harm by meddlesome practice. And whatever be the modification of the disease under consideration, it is the condition of the system at large that the physician must look to for his guide in treatment, meeting indications as they arise, and ever bearing in mind the fact, that nature has her own recuperative and conservative powers, he only playing the part of an assistant.

Louisville, Ky., March, 1852.

